# Coefficient of variation method combined with XGboost ensemble model for wheat growth monitoring

**DOI:** 10.3389/fpls.2023.1267108

**Published:** 2024-01-03

**Authors:** Xinyan Li, Changchun Li, Fuchen Guo, Xiaopeng Meng, Yanghua Liu, Fang Ren

**Affiliations:** ^1^School of Surveying and Land Information Engineering, Henan Polytechnic University, Jiaozuo, China; ^2^PIESAT Information Technology Co., Ltd, Beijing, China

**Keywords:** wheat, comprehensive growth monitoring indicators, fractional order differentiation, grey correlation analysis, XGBoost

## Abstract

**Introduction:**

Obtaining wheat growth information accurately and efficiently is the key to estimating yields and guiding agricultural development.

**Methods:**

This paper takes the precision agriculture demonstration area of Jiaozuo Academy of Agriculture and Forestry in Henan Province as the research area to obtain data on wheat biomass, nitrogen content, chlorophyll content, and leaf area index. By using the coefficient of variation method, a Comprehensive Growth Monitoring Indicator (CGMI) was constructed to perform fractional derivative processing on drone spectral data, and correlation analysis was performed on the fractional derivative spectra with a single indicator and CGMI, respectively. Then, grey correlation analysis was carried out on differential spectral bands with high correlation, the grey correlation coefficients between differential spectral bands were calculated, and spectral bands with high correlation were screened and taken as input variables for the model. Next, ridge regression, random forest, and XGboost models were used to establish a wheat CGMI inversion model, and the coefficient of determination (R^2^) and root mean squared error (RMSE) were adopted for accuracy evaluation to optimize the wheat optimal growth inversion model.

**Results and discussion:**

The results of the study show that: using the data of wheat biomass, nitrogen content, chlorophyll content and leaf area index to construct the comprehensive growth monitoring indicators, the correlation between the wheat growth monitoring indicators and the spectra was calculated, and the results showed that the correlation between the comprehensive growth monitoring indicators and the single indicator correlation had different degrees of increase, and the growth rate could reach 82.22%. The correlation coefficient between the comprehensive growth monitoring indexes and the differential spectra reached 0.92 at the flowering stage, and compared with the correlation coefficient with the original spectra at the same period, the correlation coefficients increased to different degrees, which indicated that the differential processing of spectral data could effectively enhance the spectral correlation. The three models of Random Forest, Ridge Regression and XGBoost were used to construct the wheat growth inversion model with the best effect at the flowering stage, and the XGBoost model had the highest inversion accuracy when comparing in the same period, with the training and test sets reaching 0.904 and 0.870, and the RMSEs were 0.050 and 0.079, so that the XGBoost model can be used as an effective method of monitoring the growth of wheat. To sum up, this study demonstrates that the combination of constructing comprehensive growth monitoring indicators and differential processing spectra can effectively improve the accuracy of wheat growth monitoring, bringing new methods for precision agriculture management.

## 1 Introduction

Accurately and timely understanding of crop growth is the key to regulating national agricultural structure and ensuring national food security. Remote sensing technology has the advantages of wide coverage, short revisit cycles, and low data acquisition costs, and it plays an important role in crop growth monitoring and yield estimation (Rasti et al., 2022). The commonly used methods for monitoring crop growth include artificial observation, machine vision and digital image processing, remote sensing monitoring, etc. In recent years, drone remote sensing has achieved good results in monitoring crop growth in the field because it is efficient, non-destructive, and accurate. Many scholars have adopted drone technology to conduct a series of monitoring studies on different crops (Impollonia et al., 2022; Wu et al., 2022; Nduku et al., 2023; Wu et al., 2023).

The spectra of ground objects are often affected by factors such as lighting, occlusion, climate conditions, and shooting environment. Therefore, obtaining purer spectra has always been a major concern for scholars. Fractional differentiation, as a spectral transformation method that can deeply explore the potential information of spectra, has been widely studied. For instance, Lv et al. (2021) took the tobacco SPAD (Soil and Plant Analyzer Development) value as the research object and employed the random forest method to establish a model after fractional differential pretreatment of the original spectrum, which can effectively estimate the SPAD value of tobacco; Zheng et al. (2023) calculated the spectral fractional differentiation of 0-2 orders with a step size of 0.2 and analyzed its correlation with the SPAD value of maize canopy. The results indicated that the absolute value of the correlation coefficient reached the maximum at 689nm in the 0.6 order, demonstrating that the spectral correlation with SPAD could be greatly improved after fractional differentiation treatment; Liu et al. (2020) took potatoes as the research object and calculated 0-2 order differential spectra with a step size of 0.2. They conducted a correlation analysis between differential spectrum and aboveground biomass at each order, and the results indicated that compared to integer order differentiation, fractional order differentiation can better improve the correlation between spectra and biomass. In summary, fractional differentiation has small interval changes, which can ensure slow signal-to-noise ratio transformation and provide more features for detecting certain spectral curve signals, making the model more stable.

In recent years, ensemble learning, as a new machine learning paradigm, has been widely applied to solve various regression problems by increasing the number of learners to improve their generalization ability. The XGBoost model adds regularization terms to the loss function based on the gradient lifting tree algorithm, which performs well in preventing overfitting and improving generalization ability. Liu (2022) compared four classification methods in his experiment on monitoring wheat stripe rust, and the study showed that the XGBoost model can effectively improve the accuracy of early and mid-term monitoring of winter wheat stripe rust; Li (2023) studied wheat and constructed a wheat yield prediction model based on six machine learning algorithms, including Least Absolute Convergence and Selection Operator Algorithm (LASSO), Ridge Regression (RIDGE), Support Vector Machine Regression (SVR), Random Forest (RF), Extreme Gradient Enhancement Algorithm (XGBoost), and Light Gradient Enhancement Algorithm (Light GBM). The results showed that the prediction accuracy of XGBoost model was much higher than the other five prediction models; Yang et al. (2022a) used jointing wheat as the research object and constructed a total nitrogen content inversion model under different soil fertility conditions using the XGBoost algorithm. The results showed that the XGBoost algorithm had high inversion accuracy in constructing a nitrogen content inversion model. These studies indicate that the XGBoost model has the advantages of strong generalization ability and high stability in the application of regression problems.

In the process of monitoring crop growth, single parameters such as chlorophyll content, biomass, leaf area index, and nitrogen content are often selected as key evaluation indicators. Taking rice chlorophyll content as the research object, He et al. (2023) analyzed the inversion ability of eight spectral parameters on rice chlorophyll content. The results demonstrated that the red edge area and red edge amplitude were highly correlated with chlorophyll content using rice chlorophyll content as the research object; Zhang et al. (2021) used Landsat-8 OLI images as the data sources and found that the partial least squares model, which integrates multi-band spectral information, has a good inversion effect on the aboveground biomass of vegetation crops and crops in the study area; Guo et al. (2022) employed three machine learning methods, namely, least partial quadratic regression (PLSR), support vector machine (SVM), and random forest (RF), to retrieve the wheat leaf area index. The results indicated that the combination of global sensitivity analysis and machine learning could enhance the accuracy of LAI estimation; Guo et al. (2023) used four machine learning regression methods, namely BP neural network, RF, Adaboost, and support vector machine (SVR), to invert plant nitrogen content. But using a single indicator for growth monitoring cannot accurately reflect and identify the growth status of crop populations with large morphological structures but low physiological activities, or small morphological structures but high physiological activities. Therefore, it is necessary to investigate new crop growth monitoring methods that can reflect both the level of physiological activities and the size of morphological structures. Pei (2022) used MLR, PLSR, and RF methods to construct inversion models for biomass, leaf area index, and growth monitoring indicators of different growth stages of wheat, and compared them. The results indicated that compared with the biomass and leaf area index, the comprehensive growth monitoring indicators (CGMI) can more accurately reflect the growth status of wheat; Based on the principle of equal weight, Pei et al. (2017) constructed a comprehensive index for five indicators: leaf area index (LAI), leaf chlorophyll content, plant nitrogen content, plant water content, and biomass. They combined spectral index with partial least squares regression to establish an inversion model for this comprehensive index. However, neither of them considers the contribution rate of different growth indicators to the CGMI. Simply, each indicator is evenly allocated to the CGMI. Meanwhile, considering the different importance and dimensions of each indicator in the CGMI, different weights are assigned to each indicator, and a comprehensive growth monitoring indicator is constructed based on variable weights, Xu et al. (2021) used biomass, plant height, chlorophyll content, and plant moisture content data to construct CGMI by adopting the variable weight method. The research results demonstrated that compared with a single indicator, the correlation between CGMI and spectral characteristics was improved substantially. These studies indicate that when determining the growth status of wheat, it is possible to more accurately estimate wheat growth by dividing the weights of individual growth monitoring indicators.

In this paper, to reduce background influence and improve the correlation between growth monitoring indicators and spectral features, a comprehensive growth monitoring indicator that can reflect both the physiological activity level and population morphological structure size of wheat is constructed. The fractional order differential method is employed to process the hyperspectral data of UAV, and the wheat biomass, nitrogen content, chlorophyll content, and leaf area index are used to construct the comprehensive growth monitoring index CGMI by using the coefficient of variation method. The correlation coefficient between the fractional order differential spectrum and CGMI is calculated, and the differential spectrum with a strong correlation is selected as the model input variable. Finally, ridge regression, RF, and XGboost (Extreme Gradient Boosting) models are used to construct the inversion model of the comprehensive growth monitoring index of wheat, thereby realizing the monitoring of wheat growth.

## 2 Materials and methods

### 2.1 Overview of the research area

**Figure 1:**
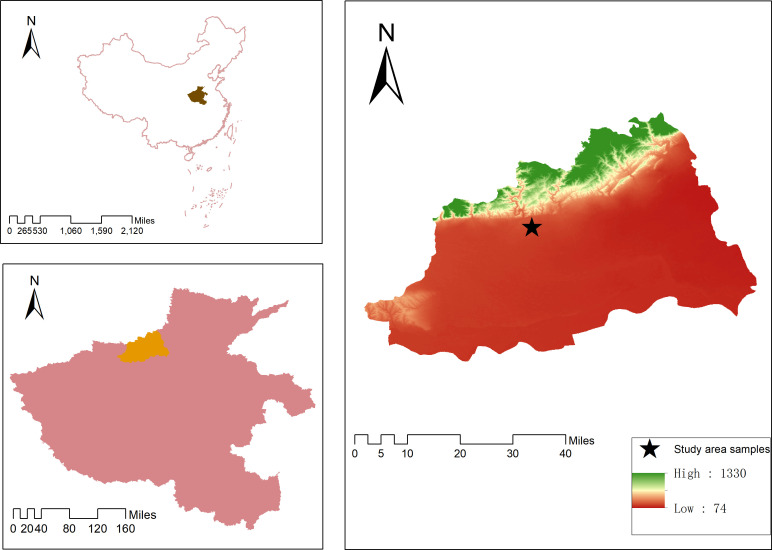
The overview of the research area.

The research area is located in the precision agriculture demonstration base of the Agricultural Science Research Institute in Jiaozuo City, Henan Province. The specific location is 35.18° N and 113.03° E. Located in the piedmont plain, the terrain is high in the west and low in the east, high in the north and low in the south, and the altitude ranges from 80 to 480 meters. The average annual temperature is 12.8 to 15.5°C, and the average annual precipitation is about 600 millimeters, belonging to a temperate monsoon climate. The experimental area consisted of 48 plots, with a single plot area of 48 m^2^ (6 m × 8 m), one replication per 16 plots; there are four fertilizer levels: 0, 195, 390, and 585 kg/hm^2^, and the rest were operated according to the actual management in the field. The specific geographical location of the study area is shown in **Figure 1**.

### 2.2 Data acquisition and preprocessing

#### 2.2.1 Drone data acquisition

Taking the eight-rotor electric UAV as the carrying platform, the Cubert UHD-185 airborne high-speed imaging spectrometer (Zheng, 2021) manufactured in Ulm, Germany was synchronously used to obtain the remote sensing data of the UAV. The camera has a spectral coverage of 454~950 nm and a spectral resolution of 4nm. With the simple characteristics of the camera and the precise characteristics of the hyperspectral, it is the lightest high-speed imaging spectrometer so far. In the jointing, flag raising, and flowering stages of wheat in 2020, data collection was performed in clear and windless weather conditions. The specific time of collection was from 11:00 to 14:00, the flight altitude was set at 50 m, and the overlap between heading and lateral directions was 80%.

The hyperspectral data processing of UAV images mainly involves radiation correction, rapid image stitching, and extraction of average spectral reflectance of small canopy. Firstly, based on the center wavelength and half wavelength width of UHD185, a radiometric calibration system was designed using MATLAB software to realize radiometric calibration from image DN values to surface reflectance. Then, the images of the survey area were filtered and processed, and the images were quickly stitched together using Agisoft PhotoScan software. Finally, in ArcGIS, the vector blocks and standard vector attribute information of several samples with uniform growth in the research community were drawn, and then IDL programming was employed to extract the average spectrum of 125 bands in each research community. In the extraction process, pixel aggregation was performed to resample the image and export it to the CSV format file for subsequent data analysis.

#### 2.2.2 Ground measurement data

##### 2.2.2.1 Leaf area index

The LAI-2200 plant canopy analyzer (Li et al., 2022) was used to collect wheat LAI data. Three sample points were randomly selected in the area with uniform growth in each plot, and the LAI values were measured four times respectively. Then, the arithmetic mean value was taken as the LAI value of the sample point. Finally, the average LAI value at three sample points was calculated as the LAI value for the cell.

##### 2.2.2.2 Chlorophyll content

Three wheat plant samples were randomly collected from areas with uniform growth in each community, and they were brought back to the laboratory. The chlorophyll content of wheat was measured using spectrophotometry (Du et al., 2017). In the measurement, a punch with a diameter of 0.8 cm was first used to remove 18 circular leaves from each leaf; then, the leaves were weighed using a balance with an accuracy of 0.001 g; finally, they were cut into fine filaments and placed in a test tube containing 95% ethanol. Next, in a sealed state, the leaves were placed in a dark environment for 7 days until they turned white. Finally, a spectrophotometer was used to measure the absorbance of the chlorophyll solution at spectral wavelengths of 655 and 649 nm, and the chlorophyll content of the leaves was calculated based on the extinction coefficient of pigment molecules at that wavelength.

##### 2.2.2.3 Nitrogen content

Synchronized with drone spectral measurement, a small number of wheat leaves from each plot were chopped and placed in an aluminum box and dried in an oven for 1 hour. Then, they were turned into an air-drying oven to dry until their quality remained unchanged. The dried samples were ground and screened. The Kjeldahl method (Sun Hye et al., 2020) was adopted to measure the nitrogen content of the wheat leaves. The calculation formula is shown in Equation 1:


(1) $$X=\frac{V_{1}-V_{2}\times c\times 0.0140}{m\times V_{3}/100}\times F\times 100\%$$

where, X is the nitrogen content, c is the solution concentration, $V_{1}$ is the volume of sulfuric acid consumed by sample titration, $V_{2}$ is the volume of sulfuric acid consumed by blank sample titration, $V_{3}$ is the volume of the digestion solution, m is the sample mass, and F is the coefficient for converting nitrogen to protein.

##### 2.2.2.4 Biomass

Wheat biomass data were obtained using a drying method (Zhu, 2022). During data collection, 10 samples were randomly selected from areas with uniform wheat growth in each community and brought back to the laboratory. After organ (stem, leaf, and ear) separation and other operations, they were placed in paper bags. When drying, the temperature was first set to 105°C for green killing (about 30 minutes), and then the temperature was set to 75°C for drying until the weight of wheat leaves no longer changed (about 48 hours). Finally, the dry mass of each organ was measured, and the biomass per unit area (kg/m^2^) was calculated based on the number of plants and tillers.

### 2.3 Research methods

#### 2.3.1 Fractional order differentiation

Differential transformation is a spectral transformation method that can improve the signal-to-noise ratio of spectral data and weaken environmental noise. Integer order differentiation is a commonly used differential transformation method, while fractional order differentiation (Wang et al., 2022) is a mathematical extension of integer order differentiation and has the advantages of “memory” and “globality”. Compared to integer order differentiation, it can explain more subtle changes and overall information in data. It mainly has three forms, namely Caputo, Riemann Liouville, and Grünwald Letnikov forms. The specific calculation formula is shown in Equation 2:


(2) $$\frac{d^{\alpha }f\left(\lambda \right)}{d\lambda^{\alpha }}\approx f\left(\lambda \right)+\left(-\alpha \right)f\left(\lambda -1\right)+\frac{\left(-\alpha \right)\left(-\alpha +1\right)}{2}f\left(\lambda -2\right)+\cdots +\frac{\Gamma \left(-\alpha +1\right)}{n!\Gamma \left(-\alpha +1\right)}f\left(\lambda -n\right)$$

where, f(λ) is the spectral reflectance; λ is the corresponding band; Γ is the gamma function; α is of any order; n is the difference between the upper and lower limits of differentiation. α = 0, 1, or 2 corresponds to the original spectrum, first-order differentiation, or second-order differentiation, respectively; When α is a decimal, it corresponds to a fractional derivative spectrum.

#### 2.3.2 Construction of comprehensive growth indicators

To obtain more accurate information on the growth status of wheat in the study area, four single indicators, namely leaf area index, chlorophyll content, nitrogen content, and biomass, were constructed as CGMI to obtain wheat growth information in the study area (Liu et al., 2022). The key issue in constructing CGMI is to determine the weight of each indicator. The traditional weighting method does not consider the contribution rate of different indicators of winter wheat to the CGMI and simply reconstructs each indicator into a comprehensive indicator based on equal weight. Considering the different importance of leaf area, chlorophyll, nitrogen content, and biomass in wheat growth monitoring, the coefficient of variation method is adopted to determine the weights of each indicator. The specific formulas can be found in Equations 3–6.


(3) $$V_{i}=\frac{\sigma_{i}}{{\bar{x}}_{i}}$$

(4) $$W_{i}=\frac{V_{i}}{\sum_{i=1}^{n}V_{i}}$$

(5) $$U_{i}=\frac{X_{i}}{max\left(X_{i}\right)}$$

(6) $$CGMI=\sum_{i=1}^{4}W_{i}U_{i}$$

where, $V_{i}$ is the coefficient of variation of the ith indicator, $\sigma_{i}$ is the standard deviation of the ith indicator, ${\bar{x}}_{i}$ is the mean of the ith indicator, ${\text{W}}_{\text{i}}$ is the weight of the ith indicator, $U_{i}$ is the normalized indicator of type i, $X_{i}$ represents the original indicator of type i, and $max\left(X_{i}\right)$ is the maximum value in the original indicator of type i.

#### 2.3.3 Modeling methods

Ridge regression, RF, and XGBoost ensemble models were selected to construct a comprehensive growth monitoring model of wheat. Ridge regression (Kai and Zhang, 2017) is a biased estimation regression method specifically designed for collinear data analysis. Essentially, it is an improved least squares estimation method that abandons the unbiased characteristic of the least squares method and obtains regression coefficients at the cost of information loss and reduced accuracy, making it a more practical and reliable regression method; RF (Jiao et al., 2021) is a sampling method based on bootstrap (self-help sampling method). It takes multiple samples from the original samples that have been put back to form a training set, uses decision tree modeling for each bootstrap sample, averages the prediction results by combining decision trees, and finally determines the final prediction results through voting. In this method, two parameters are considered: the number of decision trees and the number of segmentation nodes; XGBoost is an efficient gradient-boosting decision tree algorithm (Miao et al., 2022). As a forward addition model, its core is to integrate multiple weak learners into a strong learner using ensemble thinking. Multiple trees are utilized to make decisions together, and the results of each tree are the difference between the target value and the predicted results of all previous trees. Then, all the results are accumulated to obtain the final result, thereby improving the overall model effect.

#### 2.3.4 Model evaluation method

The coefficient of determination (Chicco et al., 2021) and Root-mean-square deviation (Hodson, 2022) (RMSE) were used to evaluate the model’s accuracy. The coefficient of determination indicates the closeness between the estimated value of the model and the measured value, and its value ranges between 0 and 1. The larger the value of ${\text{R}}^{2}$ , the higher the accuracy of the model. The specific calculation method is shown in Equation 7. The RMSE reflects the error between the estimated value of the model and the measured value. The smaller the RMSE, the higher the estimation accuracy of the model. The specific calculation method is shown in Equation 8.


(7) $$R^{2}=\frac{\sum_{i=1}^{n}{\left(y_{i}-\bar{y}\right)}^{2}}{\sum_{i=1}^{n}{\left(x_{i}-\bar{y}\right)}^{2}}$$

(8) $$RMSE=\sqrt{\frac{\sum_{i=1,j=1}^{n}{\left(x_{i}-y_{i}\right)}^{2}}{n}}$$

## 3 Results and analysis

### 3.1 CGMI construction

Taking leaf area index, nitrogen content, biomass, and chlorophyll content as single growth monitoring indicators, the coefficient of variation method was adopted to determine the weights of the four growth monitoring indicators, and then a CGMI was constructed. The calculation formulas of CGMI in the jointing, flag picking, and flowering stages are as follows, where the weights of the wheat growth monitoring indicators are represented by rounding.


(9) $${\text{CGMI}}_{\text{jointing}}=0.330U_{1}+0.180U_{2}+0.335U_{3}+0.155U_{4}$$

(10) $${\text{CGMI}}_{\text{flag picking}}=0.416U_{1}+0.136U_{2}+0.311U_{3}+0.138U_{4}$$

(11) $${\text{CGMI}}_{\text{flowering}}=0.413U_{1}+0.136U_{2}+0.311U_{3}+0.141U_{4}$$

where, U_1_ is the normalized leaf area index, U_2_ is the normalized nitrogen content, U_3_ is the normalized biomass, and U_4_ is the normalized chlorophyll content.

### 3.2 Correlation analysis

#### 3.2.1 Correlation analysis between original spectra and single growth monitoring indicators

In the jointing stage, flag-picking stage, and flowering stage, correlation analysis was performed on the denoised hyperspectral data with a single indicator, and the correlation coefficient |r| was calculated. The correlation coefficient curves for different growth stages were plotted and shown in **Figure 2A**:

**Figure 2A:**
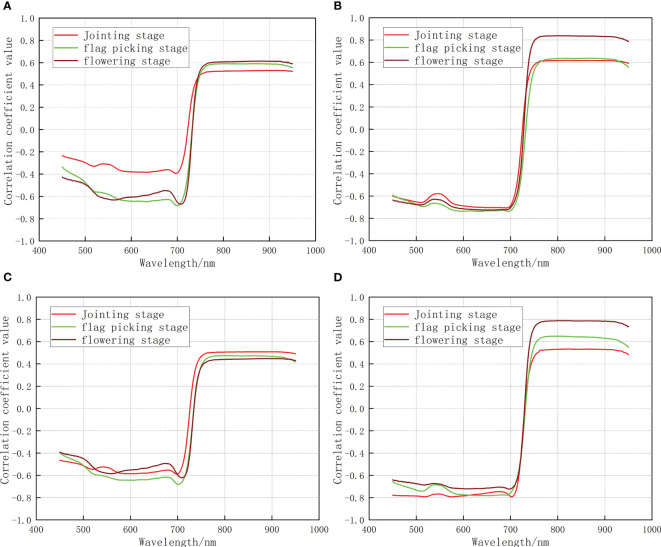
The correlation between a single growth monitoring indicator and the original spectrum **(A)** nitrogen content **(B)** biomass **(C)** chlorophyll content **(D)** leaf area index).

#### 3.2.2 Correlation analysis between original spectra and CGMI

In the jointing stage, flag picking stage, and flowering stage, correlation analysis was carried out between the denoised hyperspectral data and CGMI, and the correlation coefficient |r| was calculated. The correlation coefficient curves for different growth stages were plotted and shown in **Figure 2B**.

**Figure 2B:**
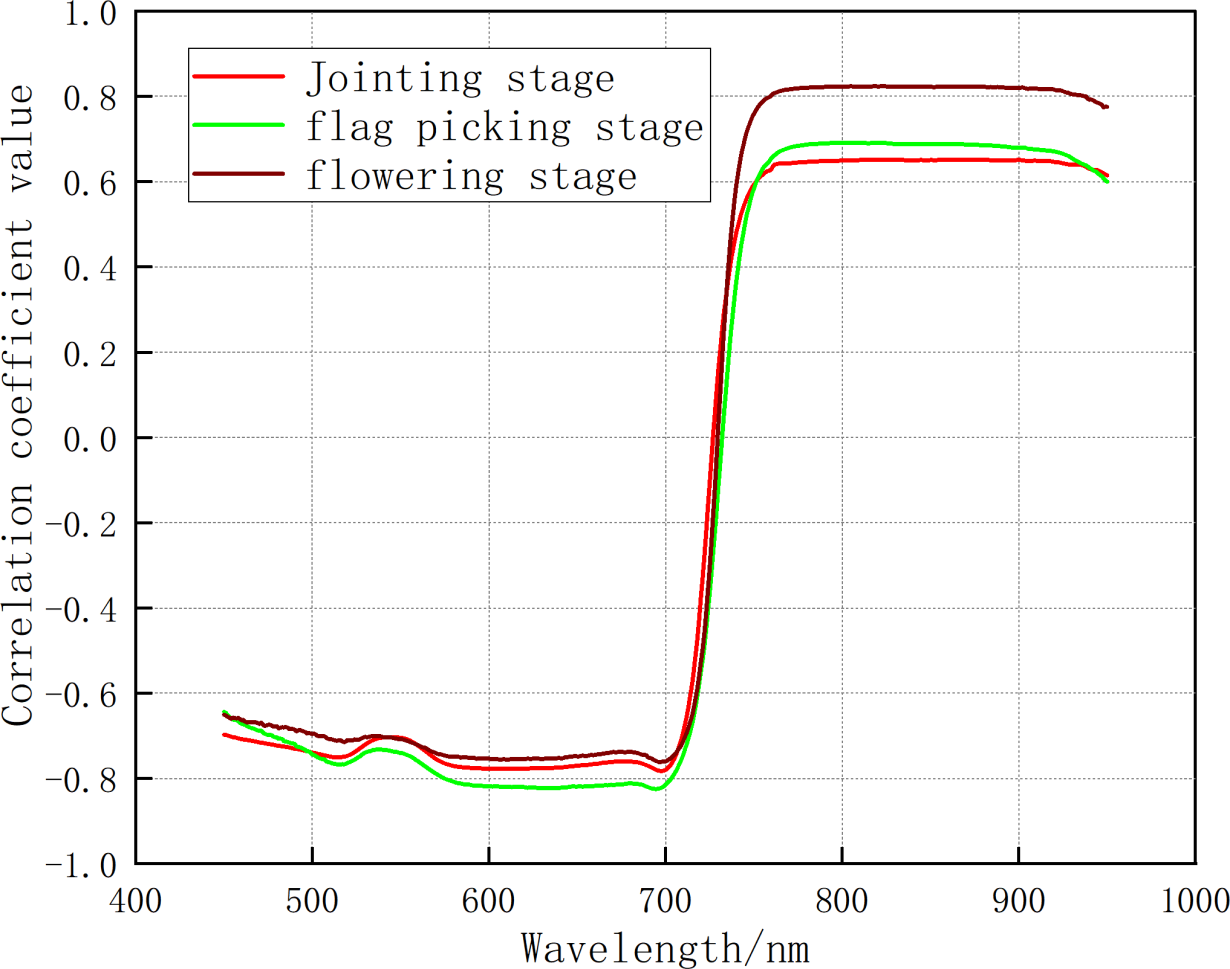
The correlation between CGMI and the original spectra.

Analysis of **Figures 2A**, **B** shows that: In the jointing stage, the maximum correlation coefficient between the original spectrum and CGMI was 0.65. It exhibited an increase of 0.12, 0.03, 0.14, and 0.12 compared to the maximum correlation coefficients between the original spectrum and nitrogen content, biomass, chlorophyll, and leaf area index, respectively. In the flag-picking stage, the maximum correlation coefficient between the original spectrum and CGMI was 0.69, with an increase of 0.10, 0.05, 0.22, and 0.04 compared to the maximum correlation coefficients between the original spectrum and nitrogen content, biomass, chlorophyll, and leaf area index, respectively. In the flowering stage, the maximum correlation coefficient between the original spectrum and CGMI was 0.82, which was lower than the maximum correlation coefficient between the original spectrum and biomass of 0.84. Compared with the maximum correlation coefficient between the original spectrum and nitrogen content, chlorophyll content, and leaf area index, there was still an increase of 0.21, 0.37, and 0.03, respectively. The above analysis indicates that CGMI can effectively improve the correlation between the original spectrum and a single growth monitoring indicator.

#### 3.2.3 Correlation analysis between differential spectroscopy and single growth monitoring indicators

Fractional order differential transformation was performed on the original hyperspectral reflectance data, with the order range set to 0 to 2 and step size set to 0.1, to obtain 20 groups of differential spectral reflectance of different orders. Then, correlation analysis was performed between nitrogen content, biomass, leaf area index, and chlorophyll content with fractional order differential spectra, and a correlation coefficient matrix diagram was drawn. The results are illustrated in **Figure 2C**.

**Figure 2C:**
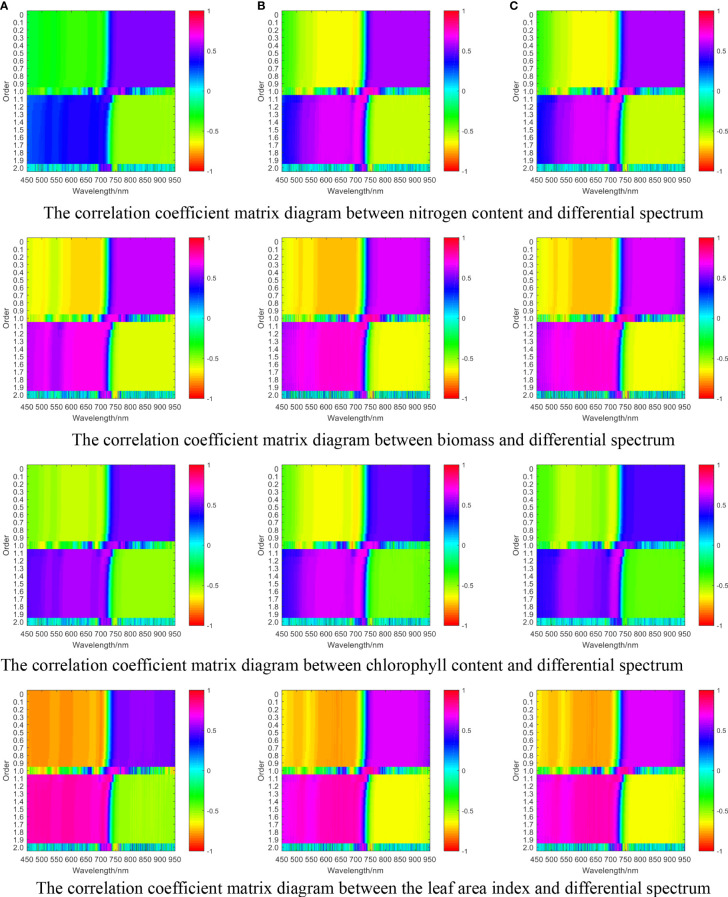
The correlation coefficient matrix diagram between single growth monitoring indicators and differential spectra of wheat **(A)** jointing stage, **(B)** flag picking stage, **(C)** flowering stage.

Comparing **Figures 2A**, **C**, it can be seen that in the jointing stage, the maximum correlation coefficients between fractional differential spectra and nitrogen content, biomass, chlorophyll content, and leaf area index were 0.65, 0.74, 0.69, and 0.81, respectively. Compared with the maximum correlation coefficients of the original spectra and the corresponding growth monitoring indicators, there was an increase of 0.12, 0.12, 0.18, and 0.28, respectively. In the flag-picking stage, the maximum correlation coefficients between fractional differential spectra and nitrogen content, biomass, chlorophyll content, and leaf area index were 0.77, 0.83, 0.73, and 0.83, respectively. Compared with the maximum correlation coefficients of the original spectra and the corresponding growth monitoring indicators, there was an increase of 0.18, 0.19, 0.26, and 0.18, respectively. In the flowering stage, the maximum correlation coefficients between fractional differential spectroscopy and nitrogen content, biomass, chlorophyll content, and leaf area index were 0.76, 0.89, 0.66, and 0.88, respectively. Compared with the maximum correlation coefficients of the original spectrum and corresponding growth monitoring indicators, there was an increase of 0.15, 0.05, 0.21, and 0.09, respectively.

Based on the above analysis, fractional differential processing of the original spectrum can effectively improve the correlation coefficient between the spectrum and growth monitoring indicators.

#### 3.2.4 Correlation analysis between differential spectroscopy and CGMI

Correlation analysis was carried out between the CGMI and fractional differential spectra, and a correlation coefficient matrix diagram was plotted. The results are shown in **Figure 2D**.

**Figure 2D:**
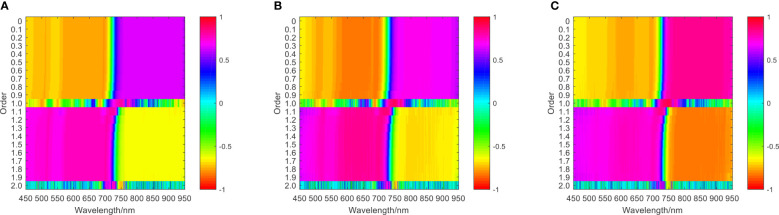
The correlation coefficient matrix of CGMI and differential spectra at different growth stages. **(A)** jointing stage **(B)** flag picking stage **(C)** flowering stage.

Comparing **Figures 2B**, **D**, it can be seen that in the jointing stage, flag-picking stage, and flowering stage, the maximum correlation coefficients between fractional differential spectroscopy and CGMI were 0.82, 0.89, and 0.92, respectively. Compared with the maximum correlation coefficients between the original spectrum and CGMI, there was an increase of 0.17, 0.20, and 0.10, respectively. Therefore, fractional differential processing of the original spectrum can effectively improve the correlation between the spectrum and CGMI.

Comparing **Figures 2C**, **D**, it can be seen that in the jointing stage, the maximum correlation coefficient between chlorophyll and fractional differential spectra was 0.69, and the bands with high correlation were concentrated in the second-order band of 700 nm. The maximum correlation coefficient between nitrogen content and the fractional-order differential spectrum was 0.65, and the bands with high correlation were concentrated in the first-order band of 774nm. The maximum correlation coefficient between leaf area content and the fractional differential spectrum was 0.81, and the bands with high correlation were concentrated around the 730nm band of 1.1 orders. The maximum correlation coefficient between biomass and fractional derivative spectra was 0.74, and the bands with high correlation were concentrated in the second-order band of 710 nm. The maximum correlation coefficient between the comprehensive growth monitoring index constructed by the coefficient of variation method and the fractional order differential band was 0.82, and the bands with high correlation were concentrated in the first-order band of 750 nm. Compared with the single growth monitoring indicators of chlorophyll, nitrogen content, leaf area, and biomass, the maximum correlation coefficients between the CGMI and the fractional order differential spectrum increased by 18.84%, 26.15%, 1.23%, and 10.81%, respectively, demonstrating a stronger correlation between the CGMI and the fractional order differential spectrum.

In the flag-picking stage, the maximum correlation coefficient between chlorophyll and fractional differential spectra was 0.73, and the bands with high correlation were concentrated around the 730 nm band of 1.1 orders. The maximum correlation coefficient between nitrogen content and fractional order differential spectrum was 0.77, and the bands with high correlation were concentrated in the first-order band of 750 nm. The maximum correlation coefficient between leaf area content and the fractional differential spectrum was 0.83, and the bands with high correlation were concentrated in the first-order band of 750 nm. The maximum correlation coefficient between biomass and fractional derivative spectra was 0.83, and the bands with high correlation are concentrated in the first-order band of 750nm. The maximum correlation coefficient between the comprehensive growth monitoring index constructed by the coefficient of variation method and the fractional order differential band was 0.89, and the bands with high correlation were concentrated in the first-order band of 750 nm. Compared with the single growth monitoring indicators of chlorophyll, nitrogen content, leaf area, and biomass, the maximum correlation coefficients between the CGMI and the fractional order differential spectrum increased by 21.92%, 15.58%, 7.23%, and 7.23%, respectively, showing a stronger correlation between the CGMI and the fractional order differential spectrum.

In the flowering stage, the maximum correlation coefficient between chlorophyll and fractional differential spectra was 0.66, and the bands with high correlation were concentrated around the 730 nm band of 1.1 orders. The maximum correlation coefficient between nitrogen content and fractional order differential spectrum was 0.76, and the bands with high correlation were concentrated in the 750 nm bands of the first and 1.1 orders. The maximum correlation coefficient between leaf area content and the fractional differential spectrum was 0.88, and the bands with high correlation were concentrated in the first-order band of 750 nm. The maximum correlation coefficient between biomass and fractional derivative spectra was 0.89, and the bands with high correlation were concentrated in the first-order band of 750 nm. The maximum correlation coefficient between the comprehensive growth monitoring index constructed by the coefficient of variation method and the differential band was 0.92. The bands with higher correlation values were concentrated in the first-order band of 750 nm, and there were 1669 bands with correlation coefficients higher than 0.8. Compared with the single growth monitoring indicators of chlorophyll, nitrogen content, leaf area, and biomass, the maximum correlation coefficients between the CGMI and the fractional-order differential spectrum increased by 39.39%, 21.05%, 4.55%, and 3.37%, respectively, indicating a stronger correlation between the CGMI and the fractional order differential spectrum.

The above analysis indicates that compared with a single growth monitoring indicator, the correlation between CGMI and differential spectroscopy has significantly increased.

### 3.3 Model variable screening

Based on the correlation analysis results, twenty highly correlated differential spectral bands were selected from each order of differential spectral bands. To prevent the strong correlation between spectral bands from affecting the stability of the model and construct a regression model with strong stability and practical significance, gray correlation coefficients were calculated for the twenty selected differential spectral bands, and the correlation degree r between elements was calculated. Then, the degree of correlation between the characteristic behavior sequence of the system and the behavior sequence of various related factors was quantified, and a matrix diagram is plotted and shown in **Figure 2E**. According to the results of the grey correlation calculation and considering the simplicity of the model, ten differential spectra with less correlation were selected as independent variables, and the CGMI was taken as the dependent variable. In the jointing stage, the 1.1th-order band of 726 nm, the first-order band of 754 nm, the 1.1th-order band of 722 nm, the first-order band of 750 nm, the 1.1th-order band of 730 nm, the first-order band of 746 nm, the 1.9th-order band of 702 nm, the 1.8th-order band of 702 nm, the 1.3th-order band of 706 nm, and the 1.2th-order band of 706 nm were selected as input independent variables; in the flag picking stage, the first-order band of 746 nm, the first-order band of 742 nm, the 1.1th-order band of 726 nm, the 1.1th-order band of 730 nm, the first-order band of 738 nm, the 1.9th-order band of 654 nm, the 1.1th-order band of 654 nm, the 1.8th-order band of 614 nm, the 1.9th-order band of 614 nm, and the 1.7th-order band of 614 nm were selected as the input independent variables; in the flowering stage, the first-order band of 754 nm, the first-order band of 746 nm, the first-order band of 730 nm, the 0.9th-order band of 866 nm, the 1.1th-order band of 730 nm, the 0.9th-order band of 854 nm, the 0.8th-order band of 866 nm, the 0.9th-order band of 822nm, the 0.9th-order band of 818nm, and the 0.7th- order band of 866nm were selected as the input independent variables.

**Figure 2E:**
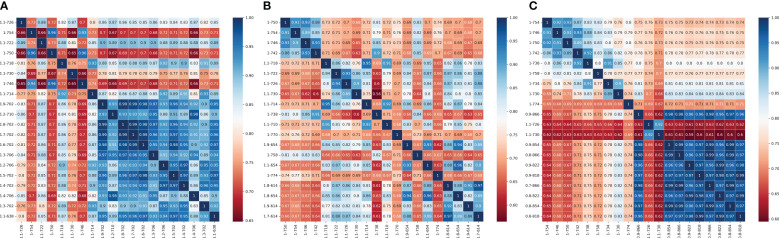
The grey correlation coefficient matrix diagram between the top 20 highly correlated differential bands screened for different growth stages. **(A)** jointing stage **(B)** flag-picking stage **(C)** flowering stage.

### 3.4 Establishment of the inversion model

#### 3.4.1 Model inversion results based on a single growth index

For different growth stages of wheat, using differential spectra as the model independent variable, 38 samples were randomly selected as the training set and 10 samples as the test set. Four estimation models for single growth monitoring indicators were constructed using three methods: random forest, ridge regression, and xgboost set model. The results are shown in **Table 1**.

**Table 1:** Inversion results of single growth monitoring indicators for different growth stages.

Growth period	Growth indicators	RF		RE		XGBOOST	
		Train	Test	Train	Test	Train	Test
**Jointing stage**	nitrogen content	0.493	0.449	0.597	0.527	0.630	0.562
	leaf area index	0.610	0.546	0.724	0.608	0.787	0.667
	chlorophyll content	0.444	0.402	0.625	0.584	0.655	0.643
	biomass	0.520	0.511	0.611	0.506	0.684	0.561
**Flag picking stage**	nitrogen content	0.631	0.615	0.755	0.717	0.720	0.645
	leaf area index	0.583	0.623	0.788	0.718	0.790	0.785
	chlorophyll content	0.535	0.511	0.643	0.602	0.684	0.614
	biomass	0.667	0.658	0.767	0.690	0.792	0.690
**Flowering stage**	nitrogen content	0.622	0.602	0.744	0.675	0.753	0.711
	leaf area index	0.721	0.659	0.838	0.747	0.858	0.753
	chlorophyll content	0.471	0.392	0.550	0.510	0.559	0.527
	biomass	0.771	0.676	0.833	0.721	0.865	0.812

Analyzing the modeling results in **Table 1**, it can be seen that the maximum R2 values of the training and testing sets for the inverted nitrogen content model are 0.755 and 0.717, the maximum R2 values of the training and testing sets for the inverted leaf area index model are 0.858 and 0.753, the maximum R2 values of the training and testing sets for the inverted chlorophyll content model are 0.684 and 0.614, and the maximum R2 values of the training and testing sets for the inverted biomass model are 0.865 and 0.812. Except for the chlorophyll content inversion model, which had the highest accuracy during the flag picking period, the nitrogen content, leaf area index, and biomass all had the highest inversion accuracy during the flowering period. Moreover, when constructing a single growth monitoring index inversion model, the XGBoost model had the best modeling effect.

#### 3.4.2 Model inversion results based on comprehensive growth monitoring indicators

By randomly sampling CGMI sample points constructed from 48 measured values, 38 samples were selected as the training set and 10 samples as the test set. Based on ten selected differential spectral bands, the CGMI of different growth stages was inverted. In the training process, the maximum number of iterations was limited to avoid the overfitting problem. To compare the accuracy of different model-building methods, RF, ridge regression, and XGboost methods were used to construct CGMI inversion models at different growth stages of wheat. The results of each model on the training set and test set are shown in **Table 2**.

**Table 2:** The inversion results of CGMI for different growth stages.

		Jointing stage		Flag picking stage		Flowering stage	
RF	Train	$R^{2}$	0.633	$R^{2}$	0.744	$R^{2}$	0.792
		RMSE	0.075	RMSE	0.076	RMSE	0.071
	Test	$R^{2}$	0.601	$R^{2}$	0.725	$R^{2}$	0.779
		RMSE	0.087	RMSE	0.081	RMSE	0.086
RE	Train	$R^{2}$	0.737	$R^{2}$	0.836	$R^{2}$	0.884
		RMSE	0.061	RMSE	0.056	RMSE	0.052
	Test	$R^{2}$	0.623	$R^{2}$	0.779	$R^{2}$	0.781
		RMSE	0.082	RMSE	0.085	RMSE	0.073
XG	Train	$R^{2}$	0.835	$R^{2}$	0.840	$R^{2}$	0.904
		RMSE	0.056	RMSE	0.063	RMSE	0.050
	Test	$R^{2}$	0.789	$R^{2}$	0.809	$R^{2}$	0.870
		RMSE	0.051	RMSE	0.060	RMSE	0.079

Analysis of the modeling results in **Table 2** shows that the accuracy of the CGMI inversion model gradually improves with the growth of the reproductive period. In the flowering stage, the accuracy of the three models on the training and testing sets are all above 0.75. In the three growth stages, the modeling results of XGboost are better than those of RF and RE. In the flowering period, the XGboost model achieves the best inversion performance, with R^2^ values of 0.904 and 0.870 and RMSE values of 0.050 and 0.079 on the training and testing sets, respectively.

Comparing the modeling results in **Tables 1**, **2**, it can be seen that during the jointing stage, the leaf area index inversion results of four single growth monitoring indicators of wheat were the best when using different modeling methods, The inversion accuracy of the comprehensive growth monitoring indicators was higher than the optimal inversion accuracy of a single growth indicator. When using the random forest method, the training and testing sets R2 increased by 3.77% and 10.07%, respectively; The R2 of the training and testing sets increased by 1.80% and 2.47% when using the ridge regression method; the R2 of the training and testing sets increased by 6.10% and 18.29% when using the XGboost method. During the flag picking period, using the random forest and XGboost models to invert the biomass of four single growth monitoring indicators of wheat showed the best results, while using the ridge regression method to invert the leaf area index showed the best results, The inversion accuracy of the comprehensive growth monitoring indicators was also higher than the optimal inversion accuracy of a single growth indicator. When using the random forest method, the training and testing sets R2 increased by 11.54% and 10.18%, respectively; The R2 of the training and testing sets increased by 6.09% and 8.50% when using the ridge regression method; the R2 of the training and testing sets increased by 6.06% and 17.25% when using the XGboost method. During the flowering period, when using the random forest and XGboost models to invert the biomass of four single growth monitoring indicators of wheat, the best results were obtained, when using the ridge regression method, the leaf area index was the best, The inversion accuracy of the comprehensive growth monitoring indicators was higher than the optimal inversion accuracy of a single growth indicator. When using the random forest method, the training and testing sets R2 increased by 2.72% and 15.24%, respectively; The R2 of the training and testing sets increased by 5.49% and 4.55% when using the ridge regression method; the R2 of the training and testing sets increased by 4.51% and 7.14% when using the XGboost method. Based on the above analysis of results, it can be concluded that when using three modeling methods to invert growth monitoring indicators at different growth stages, the modeling results of CGMI are always better than those of a single growth monitoring indicator.

## 4 Discussion

Hyperspectral remote sensing data are important for remote monitoring of crop growth. By analyzing reflectance spectral data in different bands, differences in the spectra of wheat under different fertility periods can be observed, and these differences provide rich information for growth monitoring. In order to reduce the interference of background noise on the vegetation spectra and highlight the subtle changes in the spectral curves, differential processing can be applied, which can enhance the spectral curves on the slope, thus better presenting the essential characteristics of the crop. Compared to the original reflectance spectra, the spectra after differential processing can reflect the wheat growth condition more sensitively. However, the integer order differentiation method may cause signal missing while eliminating the noise because it ignores the gradual change information of the spectrum. To solve this problem, the fractional order differentiation method can be used. Fractional order differentiation is an extension of the integer order differentiation method, which differentiates hyperspectral data at the fractional order. By using fractional order differentiation, the subtle information of the spectrum can be highlighted, the small differences between the spectral data can be described, and the absorption characteristics of the weak spectrum can be enhanced to a certain extent, so that more effective information can be retained and the effect of wheat growth monitoring can be improved. Xu et al. (2021) selected the characteristic bands of red band, green band, red edge band, and near-infrared band in the original spectrum to construct vegetation indices as model input variables, but the results were not satisfactory, and the maximum value of the correlation coefficient between their constructed vegetation indices and each growth monitoring index of wheat was only 0.580. In this paper, the fractional-order differential method was adopted to process the raw spectra, extract sensitive spectral information, and calculate the correlation between differential spectra and wheat growth monitoring indexes. The results indicated that the maximum correlation coefficient between differential spectra and different growth monitoring indexes could reach 0.92 in different growth stages, and all had different degrees of improvement compared with the original spectra, with a minimum improvement of 0.05 and a maximum improvement of 0.28. This is also consistent with the findings of Li et al. (2023).

Compared with a single monitoring indicator, comprehensively considering various growth monitoring indicators provides a new method for crop growth monitoring. As a breakthrough point, Pei et al. (2017) applied the equal weight method to construct CGMI and establish a wheat growth inversion model. However, the contribution rates of different growth monitoring indicators to wheat growth conditions inevitably differ. The researchers ignored the contribution rates of growth monitoring indicators and synthesized the indicators with equal proportions, which cannot accurately determine wheat growth. The R^2^ of the optimal inversion model is only 0.78. To address this issue, this paper proposes to use the coefficient of variation method to calculate the weights of various growth monitoring indicators for wheat at different growth stages. Following this, CGMI was constructed, and the contribution rate of each growth monitoring indicator to wheat growth was maximized. According to Equations 9–11, it can be seen that the leaf area index ranks first in the evaluation of the importance of growth monitoring, accounting for a proportion between 0.33 and 0.42, and the reason for its larger proportion may be that the leaf area index is an important index reflecting the size of the life vitality of the crop population, and within a certain range, the yield of the crop will be increased with the increase of the leaf area index. Biomass ranked second in the assessment of the importance of growth monitoring, with a proportion between 0.31 and 0.33. Biomass, as the material basis for the formation of wheat yield, is able to judge the total amount of dry matter per unit area of wheat in a given period. Nitrogen content ranked third in the assessment of the importance of growth monitoring, accounting for between 0.14 and 0.18, accounting for a smaller proportion, probably because the nitrogen in wheat is mainly present in the seeds, and is mainly expressed in the growth of roots, stems and leaves during the development of wheat, with a particularly significant effect on the increase in leaf area. Chlorophyll content ranked fourth in the assessment of the importance of growth monitoring, with a proportion between 0.14 and 0.16, probably because chlorophyll is an indicator for evaluating the photosynthetic capacity and nitrogen nutritional status of the crop, and its effect on yield is not as direct as that of the other growth monitoring indicators. Based on the results of the above weight allocation a comprehensive growth monitoring index was constructed at different fertility periods, and the four single growth monitoring indicators and the CGMI and differential spectral correlation coefficients were calculated separately. The results demonstrated that the maximum correlation coefficients between CGMI and differential spectra were 0.82, 0.89, and 0.92 in the jointing, flag-picking, and flowering stages, respectively. Compared with the maximum values of biomass, leaf area index, nitrogen content, chlorophyll content, and differential spectral correlation coefficient, the correlation coefficient increased by 10.81%, 1.23%, 26.15%, and 18.84% respectively in the jointing stage, increased by 7.23%, 7.23%, 15.58%, and 21.92% respectively in the flag-picking period, and increased by 3.37%, 4.55%, 19.48%, and 26.03% respectively in the flowering stage. Compared with the maximum correlation coefficient obtained by Pei et al. (2017) in the corresponding stages, it increased by 30.16%, 28.99%, and 29.58%, respectively. The research results indicate that the CGMI constructed using the variable weight method can effectively improve the effectiveness of wheat growth monitoring. In summary, different indicators have different impacts on the growth process of wheat, and it is necessary to allocate weights to monitor growth.

Comparing the correlation analysis and modeling results of different growth stages, the correlation between CGMI and differential spectra in the flowering stage is higher, and the modeling results are also better. In the growth process of wheat, jointing to flowering is a rapid growth period, and a large amount of nutrients is accumulated in this period. The correlation between CGMI and differential spectra shows an overall increasing trend, and the modeling results of the flowering stage are significantly better than those of the jointing and flag-picking stages. Compared with the jointing and flag-picking stages, in the flowering stage on the training and testing sets, the values of the RF model increased by 25.12%, 29.62%, and 6.45%, 7.45%, respectively; the values of the RE model increased by 19.95%, 25.36%, and 5.74%, 0.26%, respectively; the values of the XGboost model increased by 8.26%, 10.27%, and 7.62%, 7.54%, respectively. Comparing the modeling results of the three models, it is found that the XGboost model inverts CGMI with higher accuracy and better stability than the RF and RE models. Yang et al. (2022b) pointed out that the XGboost algorithm divides the original dataset into multiple subsets, and each subset randomly allocates the prediction and predicts the result according to the weight. Also, the algorithm can be parallelized to improve the running speed. As an integrated model, the XGboost algorithm is able to adapt to complex nonlinear relationships and has stronger parallel processing capability. By integrating multiple decision tree models, the overfitting problem can be reduced and the generalization ability of the model can be improved. XGboost solves the overfitting problem mainly through regularization, introducing regularization terms to control the complexity of the model and prevent overfitting. By adding regularization terms to the objective function, such as L1 regularization (Lasso) and L2 regularization (Ridge), the growth of the tree can be limited to avoid overly complex fitting. In the modeling process, to achieve high-precision monitoring of wheat growth, the fitting ability and prediction accuracy of the model is improved by adjusting hyperparameters as follows. The maximum tree depth max_depth was set to 5, and the default gamma value was set to 0. The regularization parameter lambda value controls the complexity of the model, and the greater the limit, the less likely the model is to overfit. It was set to 30. The subsample was set to 0.8 to randomly collect training samples. Besides, the learning rate eta value was set to 0.1, and the maximum number of iterations num_round was set to 100. According to the CGMI modeling results, the R^2^ values of the training and testing sets of the XGboost model during the jointing period increased by 13.30% and 26.65% respectively compared to the RE model, and 39.91% and 31.28% respectively compared to the RF model; The R^2^ values of the training and testing sets of the XGboost model during the flag picking period increased by 0.48% and 3.85% respectively compared to the RE model, and by 12.90% and 11.59% respectively compared to the RF model; The R^2^ values of the training and testing sets of the XGboost model during the flowering period increased by 2.26% and 11.40% respectively compared to the RE model, and increased by 14.14% and 11.68% respectively compared to the RF model. By using regularization techniques and adjusting parameters, the XGboost model can improve its generalization ability and ultimately achieve good estimation accuracy.

## 5 Conclusion

In this study, the precision agriculture demonstration area of Jiaozuo Academy of Agriculture and Forestry Sciences in Henan Province was taken as the research area, and the leaf area index, chlorophyll content, biomass, and nitrogen content of wheat in different growth stages were taken as the research object. A comprehensive growth monitoring index was constructed by using the coefficient of variation method, and then the differential spectrum after fractional subdivision processing was employed to build a CGMI inversion model combined with RF, ridge regression, and XGboost algorithms to effectively monitor wheat growth in the research area. By comparing the above methods, the following conclusion can be drawn: the correlation between the original spectrum and the monitoring indicators of wheat growth is relatively low, and its correlation can be greatly improved through fractional differentiation processing; Considering the different contribution rates of a single growth monitoring indicator to the comprehensive growth of wheat, the coefficient of variation method was used to construct a comprehensive growth monitoring indicator. The results demonstrated that constructing a comprehensive growth monitoring indicator can effectively improve spectral correlation; Comparing the modeling results using different modeling methods in different growth stages, the XGboost model in the flowering stage achieved the best modeling results, and it can be used as an effective method for monitoring wheat growth.

However, there are still limitations in the research that need to be overcome. The growth monitoring indicators used are not comprehensive, and plant height, vegetation moisture content, etc., also reflect the growth status of wheat. In the future, more single indicators can be incorporated to obtain more accurate and comprehensive wheat growth monitoring results; The experimental area is small, and larger experimental areas can be selected in the future to enlarge the model dataset and further verify the robustness of the model.

## Data availability statement

Due to the nature of this research, participants of this study did not agree for their data to be shared publicly, so supporting data are not available. Requests to access the datasets should be directed to XL, lxy15188234203@163.com.

## Author contributions

XL: Methodology, Software, Validation, Visualization, Writing – original draft. CL: Conceptualization, Data curation, Funding acquisition, Project administration, Writing – review & editing. FG: Writing – review & editing. XM: Writing – review & editing. YL: Formal analysis, Writing – review & editing. FR: Writing – review & editing.

## References

[B1] Chicco D. Warrens M. J. Jurman G. (2021). The coefficient of determination R-squared is more informative than SMAPE, MAE, MAPE, MSE and RMSE in regression analysis evaluation. PeerJ Comput. Sci. 7, e623. doi: 10.7717/peerj-cs.623 PMC8279135 34307865

[B2] Du Q. Wei Y. Liu P. Du H. (2017). Determination of total flavonoids in male flowers and leaves of Eucommia ulmoides by spectrophotometry. J. Cent. South Univ. Forestry Technol. 37, 96–100. doi: 10.14067/j.cnki.1673-923x.2017.05.000

[B3] Guo H. Lu Z. Xu F. Luo M. Zhang X. (2022). Estimation of winter wheat leaf area index based on global sensitivity analysis and machine learning. Zhejiang Agric. J. 34, 2020–2031. doi: 10.3969/j.issn.1004-1524.2022.09.21

[B4] Guo Y. Jing Y. Wang L. Huang J. He J. Feng W.. (2023). Prediction of winter wheat nitrogen content based on UAV image features and analysis of model migration ability. China Agric. Sci. 56, 850–865. doi: 10.3864/j.issn.0578-1752.2023.05.004

[B5] He J. He J. Wang B. Gou J. Lin Y. Liu G. (2023). Retrieval of chlorophyll content in rice based on UAV Hyperspectral imaging. J. Sichuan Agric. Univ. 1–13. doi: 10.16036/j.issn.1000-2650.202205194

[B6] Hodson T. O. (2022). Root-mean-square error (RMSE) or mean absolute error (MAE): when to use them or not. Geosci Model. Dev. 15, 5481–5487. doi: 10.5194/gmd-15-5481-2022

[B7] Impollonia G. Croci M. Blandinières H. Marcone A. Amaducci S. (2022). Comparison of PROSAIL model inversion methods for estimating leaf chlorophyll content and LAI using UAV imagery for hemp phenotyping. Remote Sens. 14, 5801. doi: 10.3390/rs14225801

[B8] Jiao Q. Sun Q. Zhang B. Huang W. Ye H. Zhang Z.. (2021). A random forest algorithm for retrieving canopy chlorophyll content of wheat and soybean trained with PROSAIL simulations using adjusted average leaf angle. Remote Sens. 14, 98. doi: 10.3390/rs14010098

[B9] Kai X. Zhang L. (2017). Application of principal component regression and ridge regression in xinjiang agricultural economy. J. Liaoning Agric. Vocational Tech. Coll. 19, 57–61. doi: 10.3969/j.issn.1671-0517.2017.01.021

[B10] Li W. Maimaiti S. Maihemuti B. (2023). Hyperspectral inversion of Soil organic matter content based on fractional differentiation. Prog. laser optoelectronics 1–12. doi: 10.3788/LOP220715

[B11] Li X. Zhou Y. Duan J. He L. Wei Y. Wang H.. (2022). Quantitative relationship between different light transmittance and leaf area index in wheat canopy. J. Wheat Crops 42, 1139–1148.

[B12] Li Y. (2023). Research on winter wheat yield prediction in Shandong by integrating multimodal data and machine learning algorithms[D] (Heilongjiang Province: Harbin Normal University). doi: 10.27064/d.cnki.ghasu.2023.001012

[B13] Liu Y. (2022). Research on wheat stripe rust monitoring methods based on non-imaging and imaging hyperspectral remote sensing[D] (Anhui Province: Anhui University). doi: 10.26917/d.cnki.ganhu.2022.000391

[B14] Liu Y. Feng H. Sun Q. Yang F. Yang G. (2020). Estimation of aboveground biomass of potatoes based on hyperspectral fractional differentiation of unmanned aerial vehicles. J. Agric. Machinery 51, 202–211. doi: 10.6041/j.issn.1000-1298.2020.12.022

[B15] Liu S. Hu Z. Han J. Li Y. Zhou T. (2022). Predicting grain yield and protein content of winter wheat at different growth stages by hyperspectral data integrated with growth monitor index. Comput. Electron Agric. 200, 4684–4703. doi: 10.1016/j.compag.2022.107235

[B16] Lv X. Xue L. Jing X. Zhang C. Xu H. Zhu Q. (2021). Estimation of SPAD values in tobacco leaves based on hyperspectral fractional differentiation. Chin. J. Agron. 37, 54–59. doi: 10.11924/j.issn.1000-6850.casb2020-0599

[B17] Miao J. Zhen J. Wang J. Zhao D. Jiang X. Shen Z.. (2022). Mapping seasonal leaf nutrients of mangrove with sentinel-2 images and XGBoost method. Remote Sens. 14, 3679. doi: 10.3390/rs14153679

[B18] Nduku L. Munghemezulu C. Mashaba-Munghemezulu Z. Kalumba A. M. Chirima G. J. Masiza W.. (2023). Global research trends for unmanned aerial vehicle remote sensing application in wheat crop monitoring. Geomatics 3, 115–136. doi: 10.3390/geomatics3010006

[B19] Pei P. (2022). Winter wheat growth monitoring based on unmanned aerial vehicle hyperspectral growth indicators. Agric. Sci. Inf. 649, 126–128. doi: 10.15979/j.cnki.cn62-1057/s2022.20.001

[B20] Pei H. Feng H. Li C. Jin X. Li Z. Yang G. (2017). UAV remote sensing monitoring of winter wheat growth based on comprehensive indicators. J. Agric. Eng. 33, 74–82. doi: 10.11975/j.issn.1002-6819.2017.20.010

[B21] Rasti S. Bleakley C. J. Holden N. M. Whetton R. Langton D. O’Hare G. (2022). A survey of high resolution image processing techniques for cereal crop growth monitoring. Inf. Process. Agric. 9, 300–315. doi: 10.1016/j.inpa.2021.02.005

[B22] Sun Hye H. Minseon K. Saerom J. Sun Y. (2020). A comparison study of crude protein contents obtained utilizing the Kjeldahl method and Dumas combustion method in foods. Anal. Sci. Technol. 33, 143–150. doi: 10.5806/AST.2020.33.3.143

[B23] Wang J. Ding J. Ge X. Zhang Z. Han L. (2022). The application of fractional differential technique in estimating soil moisture content from airborne hyperspectral data. Spectrosc. Spectral Anal. 42, 3559–3567.

[B24] Wu J. Wen S. Lan Y. Yin X. Zhang J. Ge Y. (2022). Estimation of cotton canopy parameters based on unmanned aerial vehicle (UAV) oblique photography. Plant Methods 18, 129. doi: 10.1186/s13007-022-00966-z 36482426 PMC9733379

[B25] Wu Q. Zhang Y. Zhao Z. Xie M. Hou D. (2023). Estimation of relative chlorophyll content in spring wheat based on multi-temporal UAV remote sensing. Agronomy 13, 211. doi: 10.3390/agronomy13010211

[B26] Xu Y. Cheng Q. Wei X. Yang B. Xia S. Rui T.. (2021). Unmanned winter wheat growth monitoring based on coefficient of variation method and optimized neural network. J. Agric. Eng. 37, 71–80. doi: 10.11975/j.issn.1002-6819.2021.20.008

[B27] Yang X. Yuan Z. Ye Y. Wang D. Hua K. Guo Z. (2022a). Retrieval of total nitrogen content of winter wheat based on UAV hyperspectral remote sensing. Spectrosc. Spectral Anal. 42, 3269–3274. doi: 10.3964/j.issn.1000-0593(2022)10-3269-06

[B28] Yang X. Yuan Z. Ye Y. Wang D. Hua K. Guo Z.. (2022b). Inversion of total nitrogen content in winter wheat based on hyperspectral remote sensing by unmanned aircraft. Spectrosc. Spectral Anal. 42 (10), 3269–3274.

[B29] Zhang D. Wang X. Zan M. (2021). Estimation of aboveground biomass of vegetation in the Weiku oasis based on Landsat 8 OLI images. J. Grassland Industry 30, 1–12. doi: 10.11686/cyxb2020569

[B30] Zheng X. F. (2021). Fine Classification of Typical Crops Based on UHD185 Hyperspectral Data (Jiaozuo, China: Master’s Thesis. Henan Polytechnic University). doi: 10.27116/d.cnki.gjzgc.2021.000705

[B31] Zheng Z. Chang Q. Jiang S. Fu X. Li K. Zhang Z.. (2023). Estimation of SPAD value based on UAV hyperspectral fractional differential corn. J. Northeast Agric. Univ. 54, 66–74. doi: 10.19720/j.cnki.issn.1005-9369.2023.02.008

[B32] Zhu B. (2022). Research on wheat plant height and biomass estimation based on UAV elevation data (Yangzhou, China: Master’s Thesis. Yangzhou University). doi: 10.27441/d.cnki.gyzdu.2022.000959

